# Translational Imaging Spectroscopy for Proximal Sensing

**DOI:** 10.3390/s17081857

**Published:** 2017-08-11

**Authors:** Christian Rogass, Friederike M. Koerting, Christian Mielke, Maximilian Brell, Nina K. Boesche, Maria Bade, Christian Hohmann

**Affiliations:** Section 1.4 Remote Sensing, Helmholtz Centre Potsdam—GFZ German Research Centre for Geosciences, Telegrafenberg, 14473 Potsdam, Germany; friederike.koerting@gfz-potsdam.de (F.M.K.); christian.mielke@gfz-potsdam.de (C.M.); maximilian.brell@gfz-potsdam.de (M.B.); nina.boesche@gfz-potsdam.de (N.K.B.); maria.bade@gfz-potsdam.de (M.B.); christian.hohmann@gfz-potsdam.de (C.H.)

**Keywords:** imaging spectroscopy, reflectance, geometry, geology, laboratory, hyperspectral, processing chain

## Abstract

Proximal sensing as the near field counterpart of remote sensing offers a broad variety of applications. Imaging spectroscopy in general and translational laboratory imaging spectroscopy in particular can be utilized for a variety of different research topics. Geoscientific applications require a precise pre-processing of hyperspectral data cubes to retrieve at-surface reflectance in order to conduct spectral feature-based comparison of unknown sample spectra to known library spectra. A new pre-processing chain called GeoMAP-Trans for at-surface reflectance retrieval is proposed here as an analogue to other algorithms published by the team of authors. It consists of a radiometric, a geometric and a spectral module. Each module consists of several processing steps that are described in detail. The processing chain was adapted to the broadly used HySPEX VNIR/SWIR imaging spectrometer system and tested using geological mineral samples. The performance was subjectively and objectively evaluated using standard artificial image quality metrics and comparative measurements of mineral and Lambertian diffuser standards with standard field and laboratory spectrometers. The proposed algorithm provides highly qualitative results, offers broad applicability through its generic design and might be the first one of its kind to be published. A high radiometric accuracy is achieved by the incorporation of the Reduction of Miscalibration Effects (ROME) framework. The geometric accuracy is higher than 1 μpixel. The critical spectral accuracy was relatively estimated by comparing spectra of standard field spectrometers to those from HySPEX for a Lambertian diffuser. The achieved spectral accuracy is better than 0.02% for the full spectrum and better than 98% for the absorption features. It was empirically shown that point and imaging spectrometers provide different results for non-Lambertian samples due to their different sensing principles, adjacency scattering impacts on the signal and anisotropic surface reflection properties.

## 1. Introduction

Sampling and successive analysis are basic research tasks to test and evaluate empirical relations and hypotheses. Here, different analytical instruments, which utilize various physical measurement principles provide a variety of results. Instruments that utilize the principles of spectroscopy are widely used and relate the physical responses from incident electromagnetic radiation to the physical properties of the sample. There are numerous spectroscopic approaches to retrieve the reflectance, the absorbance or the transmittance of a sample. Measured unknown sample spectra are then compared with known spectra from reference libraries to assign specific material properties.

Geoscientific applications in general and geological applications in particular often require repeatable and precise analyses of the mineralogy and elemental chemistry [1], which may be delivered by laboratory scans from imaging spectroscopy and data from other optical spectrometers. However, geoscientific applications are not the only ones, which profit from extensive laboratory studies. Ecological [2] and soil [3] related applications have benefited a lot from the integration of the laboratory scans using imaging spectroscopy to study and link, e.g., plant [2] and soil properties [3] to spectroscopic signatures. This is critical for the understanding of large areas from other imaging spectroscopy scales (UAV-based, airborne and spaceborne).

However, optical remote sensing and its near field derivative proximal sensing require reflectance as a common basis for comparisons and assignments. This is the reason why an exact processing chain for the laboratory scale is so important, as critical reference spectra for comparison may only be acquired and studied in detail in the laboratory.

The reflectance retrieval comprises illumination and scanning geometry normalization, irradiance normalization and surface reflectance anisotropies (mostly considered as Bidirectional Reflectance Distribution Function—BRDF). Any acquisition dependency of the retrieved signal has to be removed or significantly reduced during the preprocessing. For this, the sensor must be radiometrically calibrated to establish a mathematical relationship between the physical response and the measured signal. The sensor should be spectrally calibrated to enable a correct identification of material-dependent spectral features as a consequence of the materials’ atomic structure. If more than one sensor is utilized, those sensors must be optically aligned using parallel optical axes and subsequent signal processing, to provide a broader spectral range and a continuous and spatially contiguous spectrum. Modern image processing techniques are merged with spectral processing. As more and more hyperspectral imaging spectrometers become available, those hyperspectral imagers provide hundreds of quasi-continuous spectral bands per pixel and enable new applications that are not possible with traditional laboratory spectroscopic techniques. Additionally, imaging spectrometers can be mounted on various platforms such as satellites, airplanes, drones, tripods, lab racks, underwater vehicles and so forth. 

As ground samples are the ones to be mostly analyzed in our laboratory, they were also used in this work. Those were epidote, hornblende, chlorite, dolomite, illite, goethite, pyrophyllite and calcite, which are key rock forming minerals and indicator minerals in geology.

The aim is to provide a processing chain for a broad set of geoscientific applications using the VNIR 1600 and SWIR 320 m-e (400–1000 nm Visible and Near Infrared VNIR/1000–2500 nm Short Wave Infrared SWIR) imaging spectrometer system (HySPEX) that utilizes the broadly used pushbroom scanning principle as line scanner. Both sensors have different spatial resolutions. The VNIR is spatially higher resolved than the SWIR, but the SWIR provides the most relevant information for geoscientific applications as important clay minerals, carbonates and mica predominantly absorb electromagnetic energy in this spectral region [1]. The proposed processing chain focusses on at-surface reflectance retrieval and on the geometric and spectral adaptation of the VNIR to the SWIR. This can be easily reversed, because the proposed approaches are generic. The proposed processing chain for imaging spectrometers was optimized for HySPEX and is named GeoMAP-Trans. It can be adapted to any other line scanning imaging spectrometers. The proposed processing chain consists of three modules: radiometry, geometry and spectroscopy. Each module comprises several modular processing steps. In the following all steps are described in detail.

## 2. Materials and Methods

### 2.1. Materials

Proximal sensing applications are widely used and remote sensing approaches are adapted to the near field as proximal sensing methods. There are numerous manufacturers of spectrometers and imaging spectrometers. The imaging spectrometers from the Norwegian company Norsk Elektro Optikk (NEO, Skedsmokorset, Norway) were the first cross-platform systems facilitating laboratory, field and airborne usage. Other manufacturers provide similar cross-platform applications, which utilize similar sensing principles. The processing chain proposed here would be beneficial for these systems as well. In this work we focus on the HySPEX VNIR 1600/SWIR 320 m-e imaging spectrometer system owned by GFZ. The specifications are given in Table 1.

We selected geology and related samples, out of the variety of potential applications in the laboratory, because the number of geological applications and their relevance has grown significantly in the past years. Geological powder standards were selected as samples, to enable a performance evaluation of the proposed processing chain. Those samples are homogenised powders, which provide mainly diffuse reflection and may also be measured by other spectrometers. Broadly used spectrometers are, e.g., the FieldSpec 4 HiRes by PANalytical (ASD, Boulder, CO, USA) and Lambda 950 (Perkin Elmer, Waltham, MA, USA). Both are sensitive in the spectral range from 350 nm to 2500 nm. They have been used for comparison of the performance of reflectance retrieval of the processing chain. A brief summary about the spectral properties of these instruments is given in Table 2.

**Figure 1:**
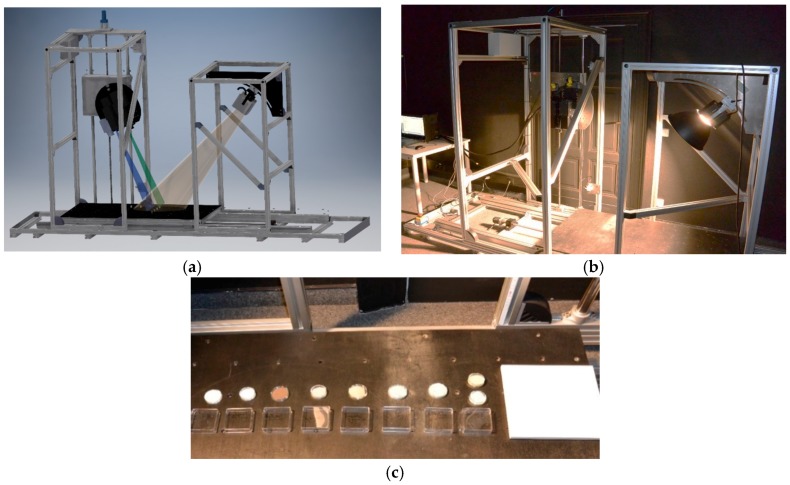
Translational imaging spectroscopy in the laboratory and the (**a**) CAD model, (**b**) experimental setup and (**c**) arrangement of the samples.

The laboratory setup is illustrated in Figure 1a,b where the sensor platform that holds all the sensors (left hand side) and the illumination mount that holds the illumination sources (right hand side) can be rotated by 90° and are mounted on moveable racks. This enables a high degree of freedom for different setups considering a wide variety of applications. The translation stage that holds the samples is electronically controlled by the HySPEX translation stage controller that is connected to the HySPEX PC and the measurement software. The software facilitates different setups including variations of the integration time, binning, averaging and stage speed. The speed of the translation stage that moves the samples through the field of view (FOV) of the imaging spectrometers is automatically adjusted to the selected integration time in order to provide a uniform ground instantaneous field of view (GIFOV), or in other words, quadratic pixels to the user.

A reflectance standard should be always placed along the sample scan direction as shown in Figure 1c. This is based on the experience from numerous measurements and the fact that low cost illumination sources tend to change their irradiance over time. That also enables the estimation of the spatial distribution of the irradiance that is necessary to estimate the at-surface reflectance of the samples as shown later. The VNIR and SWIR sensor should be mounted in parallel to enable a consistent ratio of the individual instantaneous field of views (IFOV) of both sensors, which eases the succeeding geometric co-registration. The geological samples as shown in Figure 1c are ground standards or powders having a grain size of less than 63 μm. These are from right to left two variations of epidote, chlorite, hornblende, dolomite, illite, goethite, pyrophyllite and calcite in Figure 1c. They were selected to offer a broad spectrum of important geological materials.

### 2.2. Methods

**Figure 2:**
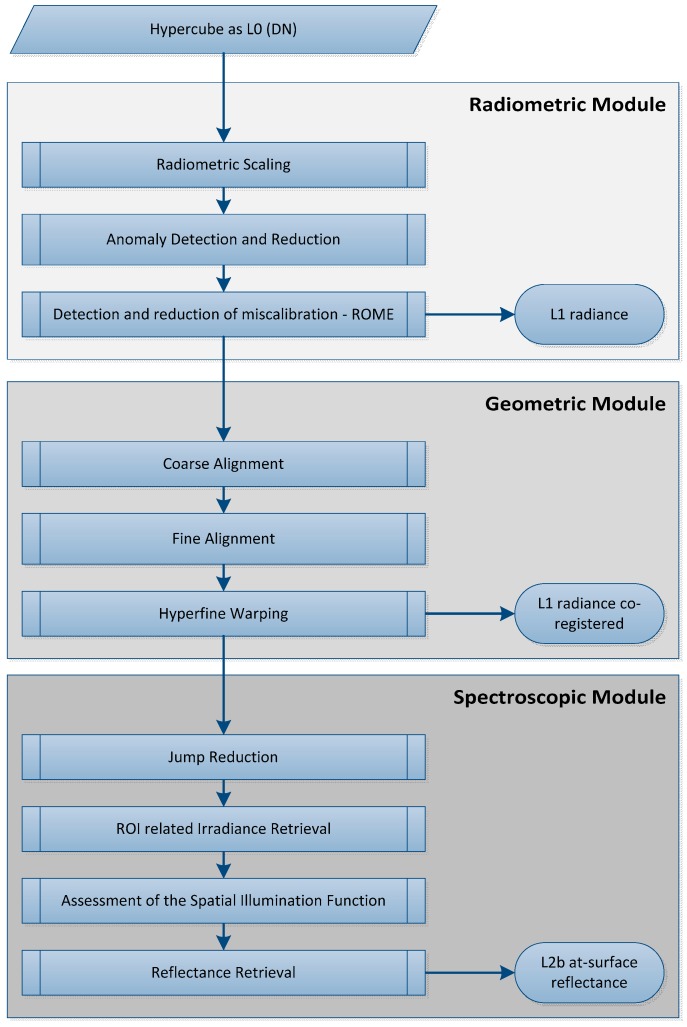
Processing scheme of the proposed approach GeoMAP-Trans.

The architecture of the proposed approach is modular. It consists of the radiometric module, the geometric module and the spectroscopic module as shown in the processing scheme in Figure 2. The modular approach has several benefits. Any module can be updated, extended or exchanged separately. This is useful if the chain contains generic approaches as the proposed approach and will be adapted to other sensors or other applications. However, the order of the modules is related to the characteristics of the different corrections and reductions, e.g., the geometric module should succeed the outlier reduction of the spectral module to avoid misregistrations based on these extreme points or the jump reduction of the spectral module should be applied after co-registration to avoid ‘de-jumping’ of not registered points that are not spatially coincident. All modules are explained in detail in the next sections.

### 2.3. Module 1: Radiometry

#### 2.3.1. Step 1.1: Radiometric Scaling

**Figure 3:**
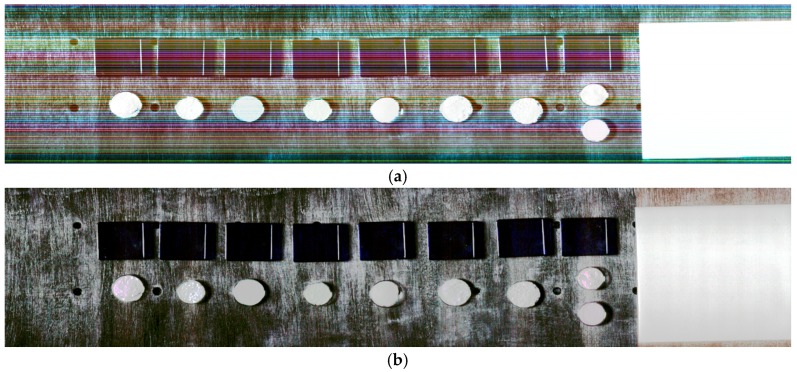
(**a**) False colour, raw HySPEX SWIR image (Red 2414.0955 nm, Green 2312.0591 nm, Blue 2210.0225 nm), (**b**) False colour, radiometrically scaled HySPEX SWIR image (Red 2414.0955 nm, Green 2312.0591 nm, Blue 2210.0225 nm).

Incident electromagnetic radiation in the visible, near infrared and shortwave infrared can be related to Digital Numbers (DNs) by the following equation, according to [6]:

(1) DN(x,λ)=NI(x,λ) · QE(λ)·SF·RE(x,λ)+BG(x,λ),

where *DN* is digital number, *x* is the across track pixel, λ the wavelength, *NI* the number of incoming photons reaching the detector matrix at position x, λ during the integration time, *QE* is the quantum efficiency of the whole system, *SF* is a scaling factor that relates *DN* to photoelectron, *RE* is the relative response matrix for each detector element and *BG* is the background matrix or dark current. Polynomial coefficients are estimated that relate recorded *DN* to radiance as physical unit of the received radiant flux in the process of radiometric calibration. Radiometric scaling balances differences on the detector characteristics and enables further analyses, as visualized in Figure 3.

A variety of different calibration approaches have been proposed throughout the years, such as vicarious calibration [7,8,9], flat field calibration [10] or laboratory calibration. The estimated coefficients are used to transform measured DN to at-sensor radiance in the process of radiometric scaling [11]. Physically well-defined calibration targets are recorded in the calibration process. Sensor response is mathematically related to the targets by polynomial modelling [12,13,14]. The data can be related to as at-sensor radiance [4,6] after radiometric scaling:

(2) $$L_{1.1}\left(x,\lambda \right)=\frac{h\cdot c\cdot \left(DN\left(x,\lambda \right)-BG\left(x,\lambda \right)\right)}{t\cdot A\cdot \Omega \cdot \Delta \lambda \left(\lambda \right)\cdot \lambda \cdot QE\left(\lambda \right)\cdot SF\cdot RE\left(x,\lambda \right)}=\frac{DN\left(x,\lambda \right)-BG\left(x,\lambda \right)}{R\left(x,\lambda \right)\cdot t},$$

where *h* is the Planck constant, *A* is the area of the entrance aperture, Ω is the solid angle of one pixel, Δλ(λ) is the spectral sampling of the camera, *t* is the integration time and R(x, λ) is the radiometric response in *DN*. Spatiospectral distortions are then contemporarily estimated using sharp spatiospectral test patterns and collimator lasers in the process of radiometric calibration. The impact of the individual distortions on successive analysis depends on the magnitude and shape of the distortions. Most relevant distortions are the ‘Smile’ or ‘Frown’ as a centre wavelength shift and the ‘Keystone’ as a band-to-band misregistration [15]. Those projection related non-uniformities have to be reduced beforehand. This is usually performed using spline interpolation. 

#### 2.3.2. Step 1.2: Anomaly Detection and Reduction

The distribution of erroneous pixels should be controlled after radiometric scaling. These changes over time may be caused by, e.g., sensor aging or dust on detector surfaces. Erroneous pixels may also be considered as spatiospectral gradients of their local pixel neighbourhood. This is valid for edges, corners and impulse noise. Edges, corners and erroneous pixels represent strong spatiospectral gradients in the x,λ-domain that can be detected using high-pass filtering techniques, in contrast to impulse noise that might be randomly distributed. At first, radiance data is averaged along-track or along the y-direction to separate random noise from object related gradients and erroneous pixel information. The along-track average is then low pass filtered using a standard boxcar filter (moving average) of size n. The result is subtracted from the along-track average. The absolute is then finally used to derive a first high pass result as given in Equation (3):

(3) $$HP\left(x,\lambda \right)=\left|{\bar{L_{1.1}\left(x,y,\lambda \right)}}_{y}-\left({\bar{L_{1.1}\left(x,y,\lambda \right)}}_{y}⊕\frac{{\underline{(1}}_{n,n})}{n^{2}}\right)\right|,$$

where HP(x,λ) is the high pass result, ${\bar{L_{1.1}\left(x,y,\lambda \right)}}_{y}$ is the along track average, ⊕ is the convolution operator in the spatial domain and $\frac{{\underline{(1}}_{n,n})}{n^{2}}$ is the two-dimensional boxcar filter of size n by *n*. This high pass result is now considered as anomaly map that contains all gradients. Assuming that object corners and object edges don’t have the same extent as the number of rows or lines (*y*) they resemble smaller gradients compared to erroneous pixels that represent contiguous, strong gradients. Numerous approaches can be applied to separate object related gradients from erroneous pixel gradients. This work uses a Laplacian approach to amplify the gradients of the high pass along track average as shown in the following equation:

(4) $$LAP\_HP\left(x,\lambda \right)=\left|\frac{\partial^{2}HP\left(x,\lambda \right)}{\partial x^{2}}+\frac{\partial^{2}HP\left(x,\lambda \right)}{\partial \lambda^{2}}\right|=\left|\Delta \left|{\bar{L_{1.1}\left(x,y,\lambda \right)}}_{y}-\left({\bar{L_{1.1}\left(x,y,\lambda \right)}}_{y}\cdot \frac{{\underline{(1}}_{n,n})}{n^{2}}\right)\right|\right|,$$

where LAP_HP(x,λ) denotes the result of the high pass filtering, ∂ denotes the differential and Δ the Laplacian operator. In addition the modulus of the Laplacian is used to relate the property of erroneous pixels to have either higher, lower or no values compared to their local neighbourhood. A hysteresis can be applied to separate erroneous pixel information from background information and to determine the pixel location of erroneous pixels after the retrieval of the absolute of the Laplacian of the absolute of the high pass along track average.

**Figure 4:**
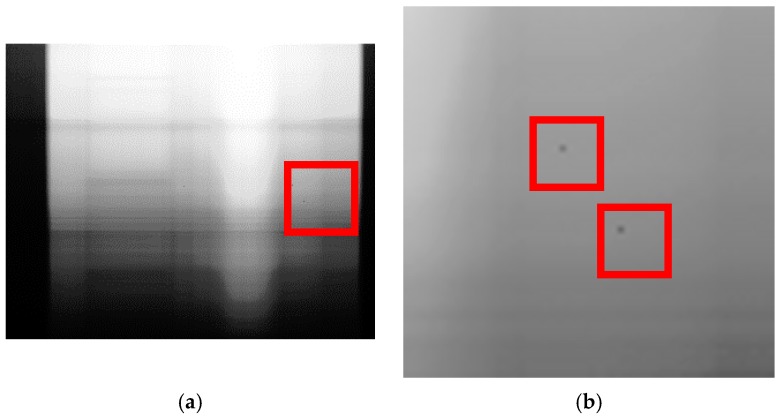

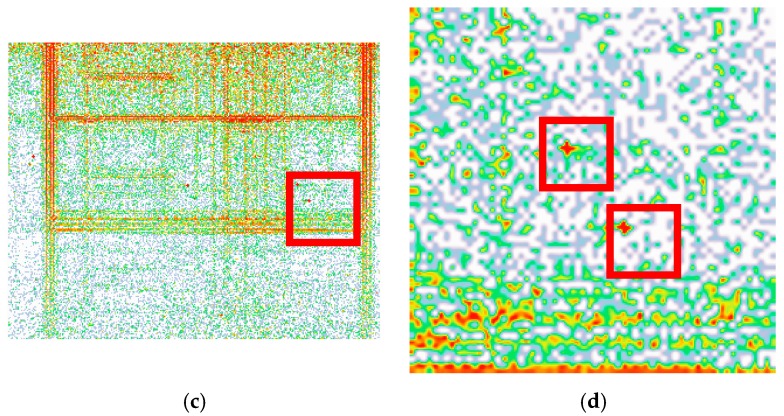
(**a**) Along track SWIR average ${\bar{L_{1.1}\left(x,y,\lambda \right)}}_{y}$; (**b**) zoom of a middle right area of (**a**); (**c**) Anomaly map LAP_HP(x,λ) and (**d**) zoom of (**c**) in the same region as in (**b**)—both erroneous detectors at *x* = 249, λ = 1699.84 nm and *x* = 260, λ = 1783.87 nm where detected as ‘anormal’ and interpolated.

In this work a threshold related to the standard deviation of the result is used for the hysteresis as given in the following equation:

(5) $$mask\left(x,\lambda \right)=\left\{\begin{array}{cc} 1 & LAP\_HP\left(x,\lambda \right)\geq factor_{1}\cdot \sigma \left(LAP\_HP\left(x,\lambda \right)\right)\\ 0 & otherwise \end{array}\right\},$$

where mask(x,λ) denotes the result and $factor_{1}$ a factor to utilize the standard deviation. The selection of 10 as $factor_{1}$ turned out to be a good compromise between detection rate of erroneous pixel and misdetection. After masking all erroneous pixels should be interpolated to avoid negative effects on successive analyses.

(6) $$L_{1.2}\left(x,y,\lambda \right)=\left(1-mask\left(x,\lambda \right)⊗\underline{1}{\left(y\right)}_{1,y,1}\right)\circ L_{1.1}\left(x,\lambda \right)+mask\left(x,\lambda \right)\circ L_{1.1,\mathrm{interpol}}\left(x,\lambda \right),$$

where $L_{1.2}\left(x,y,\lambda \right)$ denotes the result of anomaly reduction, ⊗ is the tensor or dyadic product, ∘ is the Hadamard product, $\underline{1}{\left(row\right)}_{1,row,1}$ is a tensor of 3rd order having the dimension 1 × row × 1 and $L_{1.1,\mathrm{interpol}}\left(x,y,\;\lambda \right)$ is the interpolation result. Exemplary results are visualized in Figure 4.

#### 2.3.3. Step 1.3: Reduction of Radiometric Miscalibration

**Figure 5:**
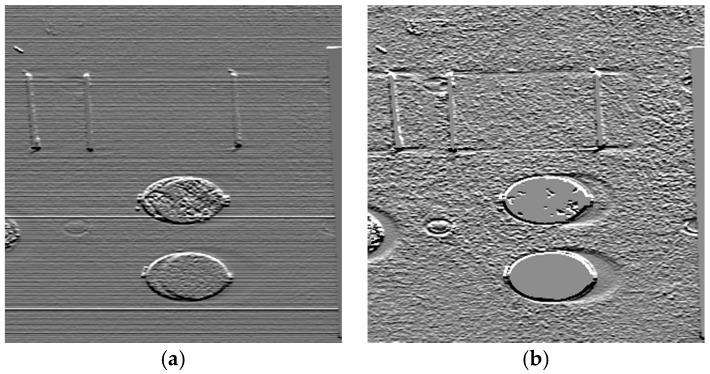
(**a**) Grey scaled image of the along track smoothed across track gradient of the first band of raw SWIR, (**b**) Grey scaled image of the along track smoothed across track gradient of the first band of radiometrically correct (perfect destriping) SWIR.

The calibration coefficients are outdated if the characteristics of the detectors are changing over time. This results in visually perceptible stripes in all processing or analysis levels. In [14,16] a framework for the—Reduction of Miscalibration Effects—(ROME) has been proposed for the efficient reduction of striping induced by miscalibration of any type. However, ROME comprises three approaches for pre-processing—linearity, non-linearity and offset correction. First, the types of miscalibrations are detected. Then, different reductions are applied step-wise, whereas the change of the Signal-To-Noise-Ratio (SNR) is evaluated after each reduction step. The processing chain is hybrid, with gain errors being detected first, then offsets and nonlinearities. The trend correction is the last step of ROME that aims at suppressing overcorrections. However, it is necessary to automatically determine the striping status. In this work Equation (6) of the approach of [13] serves as the basis for the evaluation. There, the stripes s(x,λ) are estimated as bounded across track integral of the column median of column smoothed across track gradients. The property of scene invariant along track stripes to have no along track variation is additionally utilized. Defining $L_{1.2}\left(x,y,\lambda \right)$ in the domain Ω that is a bounded domain of ${ℜ}^{3}$ gives the following equations for the evaluation of the striping status:

(7) $$\int_{\Omega }med_{y}\left(\frac{\partial \;L_{1.2}\left(x,y,\lambda \right)}{\partial x}⊕\frac{{\underline{1}}_{1,n,1}}{n}\right)dx\;\approx s\left(x,\lambda \right)\;\wedge \;si\left(\lambda \right)=\left\{\begin{array}{cc} 1 & \sigma \left(s\left(\lambda \right)\right)>factor_{2}\\ 0 & otherwise \end{array}\right\},$$

where $med_{y}$ is the along track median, si(λ) the wavelength dependent striping indicator, σ is the standard deviation and a small positive $factor_{2}$ above zero. Miscalibration produces strong gradients (compare (a) and (b) in Figure 3 and Figure 5, which are significantly superimposed on object gradients.

Acceptable evaluation results with respect to succeeding application of the approaches proposed in [14,16] are achieved using *n* = 3. The destriping is usually not necessary for this HySPEX system. However, uncorrelated offset striping may occasionally appear, which may be reduced using the offset destriping approach of [13]. This results in the computation of the destriped $L_{1.3}\left(x,y,\lambda \right)$. These rare striping artefacts may be caused, e.g., cooling disequilibrium that is thermally induced by proximal illumination source. The difference between not corrected and perfectly corrected radiometry are demonstrated in Figure 5.

### 2.4. Module 2: Geometry

This module includes the geometric alignment of all different sensors (VNIR and SWIR) in order to provide spatially contiguous and spectrally continuous pixel spectra. The optical axes of the sensors should be aligned or mounted in parallel to ensure the same Ground Instantaneous Field Of View (GIFOV) for all detector elements (considered in the following as pixels). In addition, the parallel mount reduces relative distortions of the compact support (spatial overlap between VNIR and SWIR). This enables a constant ratio of the GIFOV between all pixels of all sensors with regard to the parallel projection. However, VNIR and SWIR may have different Instantaneous Fields Of View (IFOVs), which requires an application related decision of the data resampling strategy. In relation to the selected experimental setup the IFOV of the VNIR is on average four times smaller than the SWIR, for all bands (across track ratio 1:4, along track ratio 1:2, but twice internally binned). Therefore, the first step is the down sampling the whole VNIR data set using 4 × 4 pixel aggregation (4 × 4 old VNIR pixel are averaged to one new VNIR pixel) that relates the parallel projection condition. The remaining IFOV difference is around 5% and allows for scale dependent successive processing. This is not depicted here for the sake of simplicity and compactness. The geometric alignment is realized with a three step approach: coarse, fine and hyperfine.

#### 2.4.1. Step 2.1: Coarse Alignment

The VNIR and the SWIR reference bands should cover the same wavelength range, should have the same band centres and the same spectral response function, ideally for successive relative geometry estimation [17]. Then, the compact support only differs in geometry. For our setup we selected the VNIR band 153 having the band centre at 967.584 nm as a representative band for all VNIR bands and the SWIR band 1 having the band centre at 967.578 nm as a representative band for all SWIR bands. The geometric differences between the bands of one sensor are functions of different detector (pixel) characteristics, such as, keystone, point spread function (PSF) and line-of-sight (LOS). However, the differences between spectrally and spatially adjacent detectors (pixels) are not significant for HySPEX (<micropixel) and, hence, one band can be selected as having a representative geometry for all other bands of the same sensor. The along track offset is estimated with a cross-correlation between a middle VNIR_ref_ column and a middle SWIR_ref_ column, after the coarse spatial downsampling. This can be directly performed using the approach proposed in [18], which is an FFT based algorithm that has been devised for automatic image registration. It is insensitive for affine intra-sensor deviations. A simple along track cross correlation between the middle image column of VNIR and SWIR can be applied that provides the same along track shifts as those derived utilizing the Affine Shift Theorem via the Fourier-Mellin-Transform [18,19], contrary to the utilization of the Affine Shift Theorem. A vector having a length of twice the number of rows of the SWIR plus 1 starting with the value 0 and having an interval of 1 can be used as lag, using the simple lag based cross correlation testing. The position of the highest value of the resulting cross-correlation vector gives in correspondence to the used lag vector the integer row offset between VNIR and SWIR if the experimental setup is as proposed above. The contrast dominance of the reference plate is used in both acquisitions—VNIR and SWIR. As the integer row or y-shift is estimated the whole VNIR is integer shifted towards the opposite direction that does not implicate any interpolation (binary pixel indices shifts). The lag based cross correlation approach is mostly insensitive to low SNR and low object contrast, but the Fourier-Mellin-Transform gives more precise results. Therefore, we suggest to use the lag based cross correlation for relatively short integration times and the Fourier-Mellin approach for long integration times. However, there are different sets of approaches available for coarse image alignment. After this step $L_{1.4}\left(x,y,\lambda \right)$ is retrieved, which can be considered as coarse co-registered radiance stack of VNIR and SWIR.

#### 2.4.2. Step 2.2: Fine Alignment

**Figure 6:**
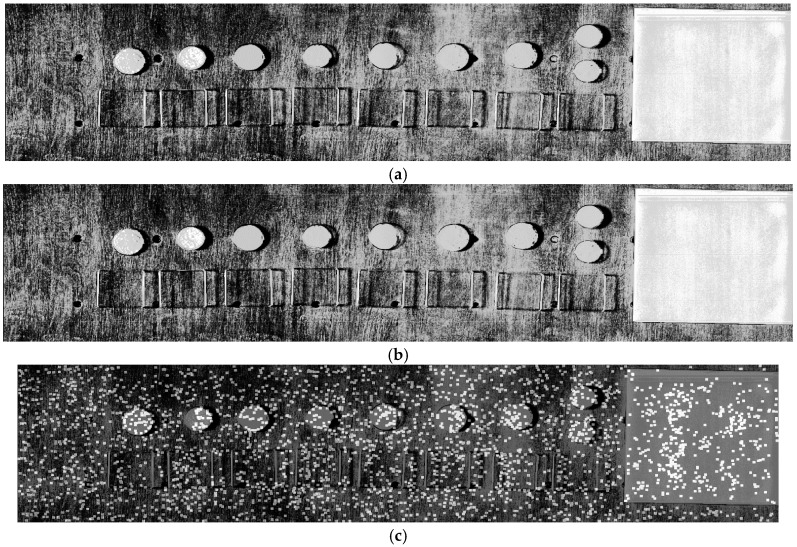
(**a**) Grey scaled VNIR band 153, (**b**) grey scaled SWIR band 1 and (**c**) VNIR band 153 as shown in (**a**), overlain by those tie points, highlighted in white, which have been found with SIFT between (**a**) and (**b**).

After step 2.1—coarse scaling and integer y-shifting - the polynomial model between VNIR_ref_ and SWIR_ref_ is estimated. The subpixel precise Scale-Invariant-Feature-Transform (SIFT, [20]) is applied for both reference images to automatically detect extreme points, whose subset serve as tie points for polynomial geometry modelling. SIFT became a standard for many image warping approaches and still is a superior algorithm with numerous implementations. In our work, the open source SIFT implementation of [21] is utilized. All extreme points are searched within a set of scaled Laplacians, selected according to their ‘extremeness’ in relation to their local neighbourhood. They are filtered according to their local contrast and described via the local gradient magnitude and direction field. Each SIFT point is then described with its subpixel coordinate and a descriptor vector that represents the local gradient neighbourhood. An exemplary result is given in Figure 6.

**Figure 7:**
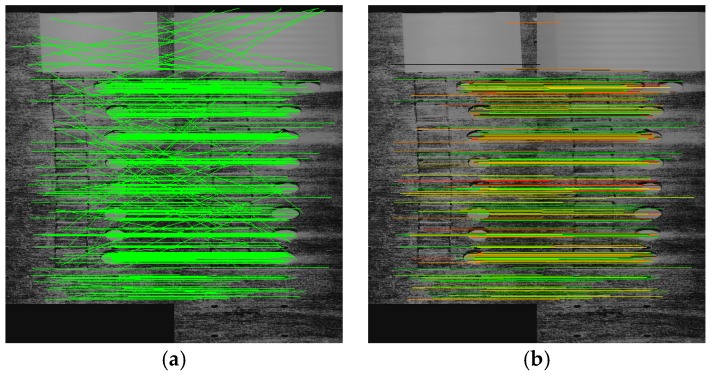
(**a**) Assigned tie point pairs after SIFT nearest-neighbour filtering between VNIR (left part) and SWIR (right part), (**b**) assigned tie point pairs after angle and iterative RANSAC filtering between VNIR (left part) and SWIR (right part).

All the resultant tie points are pairwise assigned using multiple processing sub steps. First, the Euclidean distance between the descriptors of all tie points of VNIR_ref_ and all tie points of SWIR_ref_ is computed and evaluated. Potential pairs within all combinations are found by evaluating the ratio of the smallest and the second smallest distance. If the ratio is smaller than a threshold, then the pair is assumed to be valid. We used 0.7 as the nearest neighbour threshold as recommended in [20]. Mismatches still remain (compare Figure 7a), although the previous step efficiently identified tie point pairs. Now, all angles between the y-axis and the tie point pair vector are evaluated. A tie point pair is excluded if the absolute difference between any angle and the mean angle differs more than 50% (robust upper two quartiles), related to the mean absolute difference between all angles and the mean angle. This constraint utilizes the fact that the coarse shifting of step 2.1 already suppressed the impact of the y-axis boresight, i.e., all angles should be close to 90° due to the sensor geometry. The next step re-evaluates all tie point pairs using RANSAC (the here proposed multi step outlier filtering is used to stabilize the homography solution as proposed in [22]). It can be considered as an iterative, random testing of small sets of tie point pairs for minimizing the point error of remaining tie point pairs if the currently tested polynomial model is applied. In our work, we selected a point error of 1 Pixel as stopping criterion for 1000 iterations with at least 10 points per random test set. If the point error is lower than some threshold (we used 1 pixel) the best polynomial model of RANSAC is applied on VNIR_ref_ and RANSAC is applied again with all pairs having a point error lower than the threshold. This two-step RANSAC approach efficiently filters mismatches and the average point error is used to evaluate the performance of the assessed geometric model in relation to some threshold (again 1 pixel). The multi-step outlier removal significantly improves the least squares solution of the polynomial warping model between VNIR and SWIR and is a necessary step before the successive hyperfine warping (compare Figure 7a with Figure 7b. After this step is retrieved, which can be considered as fine co-registered radiance stack of VNIR and SWIR.

#### 2.4.3. Step 2.3: Hyperfine Warping

**Figure 8:**
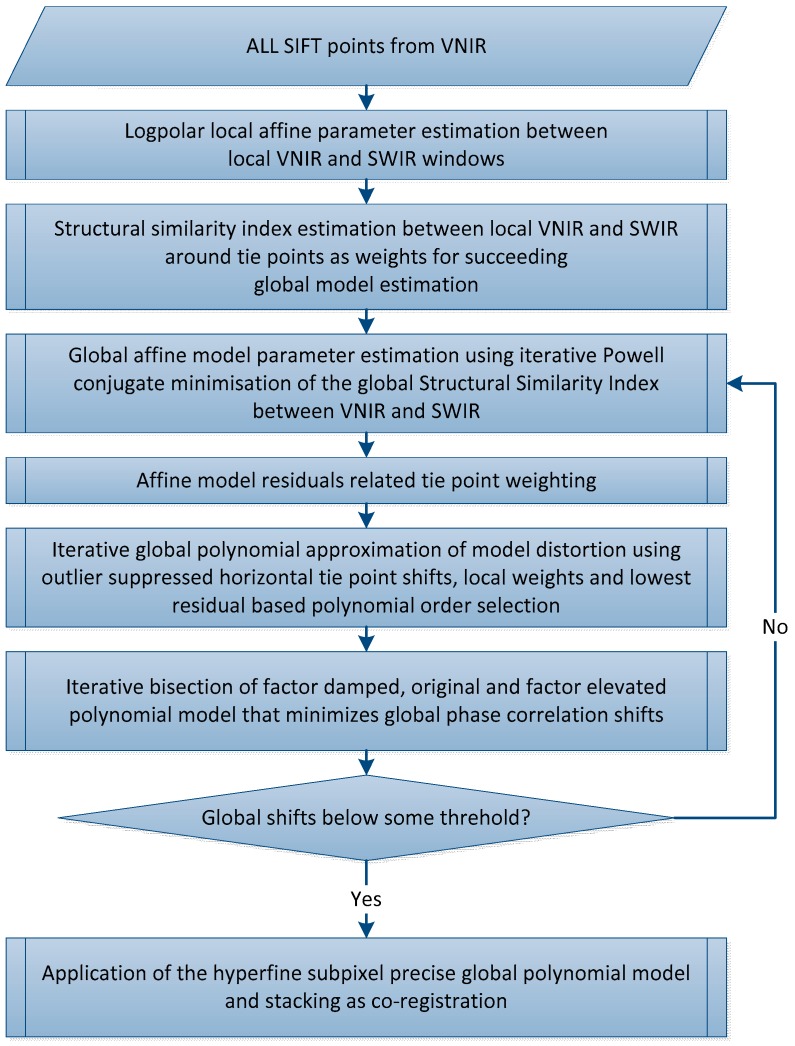
Scheme for hyperfine SIFT based logpolar warping.

Subpixel accuracy will be additionally improved after applying the global polynomial model of the previous step. This is necessary, because within SIFT the subpixel coordinate is assessed in the distorted image that may consist of global and local distortions. A subpixel precise keystone estimation technique of Rogass et al. [23] was adapted for spatial distortion estimation, which additionally minimizes the micro pixel precise phase correlation between local warp and image sample as proposed in Rogass et al. [24]. This is used to overcome this limitation of SIFT and to improve the subpixel accuracy. However, the hyperfine warping approach consists of multiple steps, which are schematically represented in Figure 8.

First, local affine distortions are estimated around all remaining SIFT points of the previous filtering step. The image content inside a small window of an arbitrary size (we use 32 × 32 pixels) centred at the individual tie point position is extracted from the VNIR_ref_ and the SWIR_ref_. Then, both image subsets, in the following named as Warp and Ref, are transformed from the row-column-domain to the logpolar domain as proposed in Xie et al. [18]. In Xie et al. [18] the affine shift theorem is utilized by transforming the image content to the logpolar domain. There, the x- and y-shifts in the logpolar domain are estimated using cross correlation that represents scale and angle, if the assessed logpolar x-y-shifts are inverted using a logpolar transformation. Then, the angle and the scale distortion is suppressed using bicubic interpolation and the global linear x- and y-shifts are estimated using cross correlation.

In Rogass et al. [24] an approach has been proposed that achieves a significantly higher accuracy for global shifting estimation than the classic cross correlation. This iterative phase correlation approach is not limited according to its application domain and, hence, can be also applied in the logpolar domain to substantially increase the accuracy of this processing step for angle and scale assessment. This achieves micro pixel accuracy also for local affine distortions. The approach of Foroosh et al. [25] can be also utilized if processing time is more important than accuracy. After logpolar affine parameter estimation individual weights are estimated for each tie point, which are then used as a priori weights of succeeding least squares global affine parameter estimation. 

The modified structural similarity index [26] between the local, SIFT point related VNIR window and the local, corresponding SIFT point related SWIR window is then estimated and normalized to the floating point range between 0 and 1 as SIFT point weight.

In a next step the first global affine parameter estimation will be conducted using all local affine parameters, the corresponding weights and an iterative Powell conjugate minimisation [27]. The empirical standard deviations of graduated observations (a posteriori residuals of the local affine parameters) are the weights for the succeeding global polynomial model approximation that is used to model high order distortions that are not covered by affine models. The best model is selected by evaluating the empirical standard deviation for different polynomial orders during polynomial modelling. The polynomial model that performs best is selected for succeeding fine adaption of the polynomials. For this global factors higher than 1, equal to 1 and lower than one are selected as starting scaling factors of bisectional polynomial modelling. The same technique is used for keystone assessment as proposed in [23] and iteratively improves global polynomial modelling. This is necessary for high order polynomials that tend to overcompensate data trends if outliers are not fully excluded in the polynomial modelling process. Each iteration of the bisection automatically adapts the three scaling factors (upper, middle, lower) with regard to assessed trends of in parallel estimated global shifts [24]. Consequently the model with the lowest remaining shifts is selected as the preliminary co-registration model, if linear and nonlinear distortions are efficiently reduced due to some threshold. If the shifts are above the threshold, then the empirical standard deviations of graduated observations of the models are used as weights for the global affine parameter estimation to iterate again. The final model for co-registration is applied, VNIR and SWIR are spectrally continuously stacked to $L_{1.6}\left(x,y,\lambda \right)$ after all previous steps. The final model consists of all three assessments—coarse, fine and hyperfine, but only one data resampling is applied. All other resampling was only applied temporary.

### 2.5. Module 3: Reflectance Retrieval

The starting product level for this module is the co-registered radiance that will be transformed by this module into at-surface or at-object reflectance. At-surface reflectance can be considered as a ratio between reflected and incident radiation (irradiance) and, hence, is considered to be highly independent of illumination effects and sensing geometry. This is a necessity for succeeding analyses, which usually rely on a statistical comparison between unknown pixel spectra and known library spectra. Several processing steps are necessary, to retrieve reflectance. In a first step a jump correction is applied to suppress the impact of different quantum efficiencies of the sensors in overlapping spectral regions and to suppress the influence of the boresight of both sensors. The boresight impact is significantly higher than the quantum efficiency differences. Due to the imperfect parallel alignment in the micro pixel scale at sharp object boundaries the line-of-sight (LOS) of one VNIR detector may cross an edge and its corresponding, co-registered SWIR detector may miss the same edge. This is perceptible as ‘jump’ in an edge spectrum if VNIR and SWIR were spectrally continuously stacked beforehand as suggested above. 

#### 2.5.1. Step 3.1: Jump Reduction

However, the jump correction can be considered as an approach to provide smooth spectra in the spectral regions of the detectors which are lowered in their sensitivity (the jump correction is only necessary for non-flat surfaces). Here, we only want to correct spectra that are from the same target and may be incorrect due to micro structure shadowing caused by different LOS. Therefore, we have to select one sensor as reference and the other sensor for correction. In this work the SWIR sensor was selected, because its average FOV is about four times higher than the average FOV of the VNIR in the measurement mode ‘ground’ and with lenses for 30, 100 and 300 cm distance. Therefore, it’s more likely that the SWIR sensor is affected by shadowing than the VNIR sensor through its coarser spatial resolution. If we assume the following relation with regard to an adaption of a definition given by [28]:

(8) $$L_{1.6,\mathrm{direct}}\;=\;\frac{\rho_{1}}{\pi \cdot \left(1-S\cdot \rho_{1}\right)}\cdot \left(\tau_{direct}\cdot E_{direct}\cdot \mu_{il}+\;\tau_{diffuse}\cdot E_{diffuse}\right)+L_{Path_{1.6}},$$

(9) $$L_{1.6,\mathrm{diffuse}}=\;\frac{\rho_{1}}{\pi \cdot \left(1-S\cdot \rho_{1}\right)}\cdot \;\tau_{diffuse}\cdot E_{diffuse}+\;L_{Path_{1.6}},$$

(10) $$\underset{d\to 0}{\lim}L_{Path_{1.6}}=\;0\;,$$

where $L_{1.6,\mathrm{direct}}=L_{1.6,\mathrm{direct}}\left(x,y,\lambda \right)$ is the integrated scaled radiance acquired from the VNIR sensor in band 153 (λ = 967.58 nm), $L_{Path_{1.6}}=L_{Path_{1.6}}\left(x,y,\lambda \right)$ is the integrated path radiance directly scattered into the sensor that becomes zero for zero distance *d*, $\rho_{1}=\rho_{1}\left(x,y,\lambda \right)$ is the integrated reflectance of the object in the inspected wavelength range, $E_{direct}=E_{direct}\left(x,y,\lambda \right)$ is the integrated direct irradiance, $E_{diffuse}=E_{diffuse}\left(x,y,\lambda \right)$ is the integrated diffusive radiance, $\tau_{direct}=\tau_{direct}\left(\lambda \right)$ the direct transmittance, $\tau_{diffuse}=\tau_{diffuse}\left(\lambda \right)$ is the diffuse transmittance, *S* is the background albedo (every possible surface was painted black in the laboratory to have a very small *S*), $\mu_{il}$ is the cosine of the illumination zenith angle (mostly used 45° as illumination zenith angle to avoid shadowing by the cameras and assumed to be constant for small object dimensions) and $L_{1.6,\mathrm{diffuse}}=L_{1.6,\mathrm{diffuse}}\left(x,y,\lambda \right)$ is the integrated scaled radiance acquired from the SWIR sensor in band 1 (λ = 967.58 nm) in the shadow. If we want to reduce the shadow impact on $L_{1,6,\mathrm{diffuse}}$ the unknown $\rho_{1}\cdot \tau_{direct}\cdot E_{direct}\cdot \mu_{il}$ must be added, which also reduces the impact of the missing hemisphere for diffuse illumination caused by the shadow casting object. The solution is the multiplication of $L_{1,6,\mathrm{diffuse}}$ with the ratio $\frac{L_{1.6,\mathrm{direct}}}{L_{1.6,\mathrm{diffuse}}}$. This ratio is biased by the geometric impact of the keystone, which is often significantly higher for first and last bands of one sensor and by the different spectral response functions of spectrally similar VNIR and SWIR bands that has to be adjusted beforehand [29]. It is recommended to reduce the jumps from band to band of the sensor to provide a smooth solution. In our work we experienced best results for three successive bands after stacking—old band 152 to old band 153, then new band 153 to old band 154 (λ = 973.58 nm) and finally new band 154 to old band 155 (λ = 979.58 nm). Hence, the first step for reflectance retrieval is the jump reduction. The new band 153 is then removed from the stack to avoid having two bands centred at 967.58 nm. After jump correction $L_{1.7}\left(x,y,\lambda \right)$ is retrieved as jump-corrected, co-registered radiance stack.

#### 2.5.2. Step 3.2: ROI Related Irradiance Retrieval

After the jump reduction of step 3.1 the spatial function of the integrated total irradiance $E_{total}=E_{direct}\cdot \mu_{il}+\;E_{diffuse}$ has to be estimated. In most cases scientific work requires a variety of different setups. However, this also means that reference plates are not fixed and often placed according to the individual setup needs. Therefore, it is recommended either to work always with a fixed, or a scenario setup, or to draw regions of interests (ROI) for masking the plates in the image data cube. A high degree of freedom was preferred in our applications, which results in the demand for providing a ROI for each setup. Using a ROI average column spectra of the reference plate can be computed as:

(11) $$Lib_{irr,1}\left(x,\lambda \right)=\frac{{\left(\left(mask_{roi}\left(x,y\right)⊗{\underline{1}}_{1,1,\lambda }\left(\lambda \right)\right)\circ L_{1.7}\left(x,y,\lambda \right)\right)}^{T}{\underline{1}}_{1,y,1}\left(y\right)}{{\left(mask_{roi}\left(x,y\right)⊗{\underline{1}}_{1,1,\lambda }\left(\lambda \right)\right)}^{T}{\underline{1}}_{1,y,1}\left(y\right)},$$

where $Lib_{irr,1}\left(x,\lambda \right)$ is the matrix of the row averaged irradiance on the reference plate, $mask_{roi}\left(x,y\right)$ is the binary mask of the ROI having the same dimensions as one band of $L_{1.7}\left(x,y,\lambda \right)$ that is co-registered radiance data cube or tensor of 3rd order from the previous jump reduction step, ⊗ is the tensor or dyadic product, ∘ is the Hadamard product, ${\underline{1}}_{1,1,\lambda }\left(\lambda \right)$ a tensor of 3rd order having the dimensions 1 × 1 × bands valued with 1, ${\underline{1}}_{1,y,1}\left(y\right)$ is a tensor of 3rd order having the dimension 1 × row × 1. A boxcar (moving window) smoothing is applied to suppress outliers as given in the following:

(12) $$Lib_{irr,2}\left(x,\lambda \right)=Lib_{irr,1}\left(x,\lambda \right)*fil_{1},$$

where $Lib_{irr,2}\left(x,\lambda \right)$ is the smoothed matrix of the row averaged irradiance on the reference plate, * the convolution operator and $fil_{1}$ a two-dimensional matrix valued with 1/n and having the dimensions of *n* × 1 (we used *n* = 5 in this work). After this the smoothed plate irradiance is normalised with the absolute reflectance of the reference plate to retrieve absolute reflectance for the whole data cube in later steps. This is performed like given in the following:

(13) $$Lib_{irr,3}\left(x,\lambda \right)=\frac{Lib_{irr,2}\left(x,\lambda \right)}{{\left({\underline{1}}_{1,x}\left(x\right)⊗\;\rho_{plate,\;abs}\left(\lambda \right)\right)}^{T}},$$

where $Lib_{irr,3}\left(x,\lambda \right)$ is the normalised, smoothed plate irradiance, ${\underline{1}}_{1,x}\left(x\right)$ is a matrix valued with 1 and having the dimensions 1 × column and $\rho_{plate,\;abs}\left(\lambda \right)$ is the average absolute reflectance spectrum of the reference plate. 

#### 2.5.3. Step 3.3: Assessment of the Spatial Illumination Function 

In the next step a smoothed, low order polynomial is found to estimate an irradiance function for the full swath of the scan that is not covered by the plate ROI in most cases. This is performed using polynomial regression as given in the following:

(14) $$Lib_{irr,4}\left(x,\lambda \right)={\underline{X}}_{1}{\left({\underline{X}}_{2}{}^{T}{\underline{X}}_{2}\right)}^{-1}{\underline{X}}_{2}{}^{T}\;{\left(vec\;(Lib_{irr,3}\left(x,\lambda \right))\right)}^{T},$$

where $Lib_{irr,4}\left(x,\lambda \right)$ is the polynomial fit of $Lib_{irr,3}\left(x,\lambda \right)$, ${\underline{X}}_{1}$ is the Vandermonde matrix of the m+1 column products of a row vector that holds all possible column indices and m is the polynomial fitting degree, ${\underline{X}}_{2}$ is the Vandermonde matrix of the *m* + 1 column products of a row vector that holds all possible column indices of the ROI and *vec*() is the vectorisation function of $Lib_{irr,3}(x,\lambda$). An iterative region growing within the reference plate ROI starts that aims on delineating the spatial extent of the reference plate to improve the assessment of the illumination function in the last step after the illumination function has been estimated using the equation for $Lib_{irr,4}\left(x,\lambda \right)$. For this, the spectral and spatial low pass LP(x,y,λ) of the co-registered, jump corrected $L_{1.7}\left(x,y,\lambda \right)$ is estimated as convolution result between $L_{1.7}\left(x,y,\lambda \right)$ and a 3D boxcar filter $\frac{{\underline{(1}}_{n,n,n})}{n^{3}}$ of size n × n × n as given by the following equation:

(15) $$LP\left(x,y,\lambda \right)=L_{1.7}\left(x,y,\lambda \right)⊕\frac{{\underline{1}}_{n,n,n}}{n^{3}}.$$

Then, a synthetic panchromatic band is assumed to be estimated as the equivalence of the sum over bands for hyperspectral instruments (small bandpass) as given by the following relation:

(16) $$PAN\left(x,y\right)=\int LP\left(x,y,\lambda \right)\cdot SRF_{PAN}\left(\lambda \right)d\lambda \;\propto \sum_{bands}LP\left(x,y,\lambda \right),$$

where PAN(x,y) is the panchromatic band that covers the full wavelength range of $L_{1.7}\left(x,y,\lambda \right)$ and $SRF_{PAN}\left(\lambda \right)$ is an estimated Gaussian shaped Spectral Response Function (SRF) that covers the full wavelength range of $L_{1.7}\left(x,y,\lambda \right)$. It appears that the equivalency of relation (16) holds for all sensors where the FWHMs of all bands are not significantly higher than the band centre differences. Statistical tests showed that for a Gaussian shaped FWHM ≤2 bands the mean absolute error does not exceed 1%. After pan retrieval a region growing [30] is conducted that utilizes the user-given ROI and the PAN. The maximum and the minimum grey value within the ROI of the PAN are selected as hysteresis threshold. This is used as a new mask and the spatial illumination function is computed using Equations (11)–(14). Missing columnar data of the new mask is substituted with $Lib_{irr,4}(x,\lambda$) beforehand. This improves the assessment if data is missing. The final illumination or irradiance function is then denoted as $Lib_{irr,5}\left(x,\lambda \right)$.

#### 2.5.4. Step 3.4: Reflectance Retrieval

After irradiance retrieval for the given ROI and the region growing derived ROI the following equation is used to automatically determine the appropriate irradiance function:

(17) $$Lib_{irr}=\{\begin{array}{cc} corr\left(\bar{Lib_{irr,4}\left(ROI_{user}\right)},\bar{Lib_{irr,5}\left(ROI_{region\;growing}\right)}\right)\text{>}threshold, & Lib_{irr,5}\\ otherwise, & Lib_{irr,4} \end{array},$$

where *corr()* denotes the correlation coefficient between −1 and 1. If the correlation coefficient between the mean spectrum of the user ROI and the mean spectrum of the region growing ROI is higher than some *threshold*, then either the irradiance function $Lib_{irr,5}$ or $Lib_{irr,4}$ is used for reflectance retrieval as irradiance. Assuming that the background albedo is close to zero through special laboratory preparation. The total atmospheric absorption is close to zero and the path radiance does not significantly contribute within one or two meters distance between sensor and illumination source, Equation (8) can be simplified to:

(18) $$\rho \left(x,y,\lambda \right)=\;\frac{L_{1.7}\left(x,y,\lambda \right)\cdot \pi }{Lib_{irr}\;\left(x,\lambda \right)⊗{\underline{1}}_{1,y,1}\left(y\right)},$$

where ${\underline{1}}_{1,y,1}\left(y\right)$ is a row vector of the length of number of rows and valued 1. 

## 3. Results and Discussion

The performance of the proposed approach will be evaluated by different criteria. Those are local and global parameters for the geometry. The geometric criteria are:

- **dx** pixel shift,
- **dy** pixel shift,
- rotation **angle**,
- scale,
- MSSIM a priori and
- MSSIM *a posteri*.

The MSSIM as proposed in [31] is the modified structural similarity image measure and can be considered as an universal image metric to measure the similarity of two images. Although it is not possible to have a MSSIM of 1 between VNIR and SWIR due to the different physical properties of both sensors, a MSSIM close to 1 (100% similarity) indicates an equivalent radiometry and geometry. The spectral part of the processing chain was evaluated using two types of metrics. The first type is objective and relates processed plate image spectra and NIST calibrated library plate spectra. The second type is subjective and relates processed sample image spectra and sample spectra of other spectrometers. Therefore, the following metrics were used to evaluate the quality of the spectral module:

- Correlation coefficient between NIST library plate spectra and processed plate image spectra of the ROI named as *Spectral Deviation Reference Plate ROI.*
- Correlation coefficient between NIST library plate spectra and processed plate image spectra of the region growing derived area names as *Spectral Deviation Reference Plate region growing.*
- Variation of the processed plate image spectra of the region growing derived area named as *Spectral Variation Reference Plate.*
- *Full spectrum correlation coefficients* between the sample spectra of ASD and Perkin Elmer (for overview), between ASD and HySPEX, between Perkin Elmer and HySPEX.
- *Continuum removed spectrum correlation coefficients* between the sample spectra of ASD and Perkin Elmer (for overview), between ASD and HySPEX and between Perkin Elmer and HySPEX.

The radiometry is not evaluated, because the number of erroneous pixels for the HySPEX system were not significant. A miscalibration was not detectable for the GFZ system and the estimation of the calibration coefficients is not part of the processing chain (only possible in special calibration facilities).

### 3.1. Geometry

The complexity of the geometric part of the processing chain dominates. This geometric part is used for the registration of VNIR and SWIR with successive image stacking without any orthorectification, which is barely required in the laboratory. The SIFT tie points served as evaluation location. First, a window having a pixel size of 16 × 16 were centred around the SIFT point and the local image content was extracted from band 152 of the resampled VNIR and band 1 of the SWIR (now band 153 of the stack). Then, the local angle, scale, dx and dy shifts were estimated using the proposed hybrid phase correlation approach in the log-polar domain. This is repeated for each SIFT point and the results are averaged. To suppress the impact of potentially remaining outliers, the 3-sigma confidence interval was only used for averaging that is given in the following Table 3.

**Figure 9:**
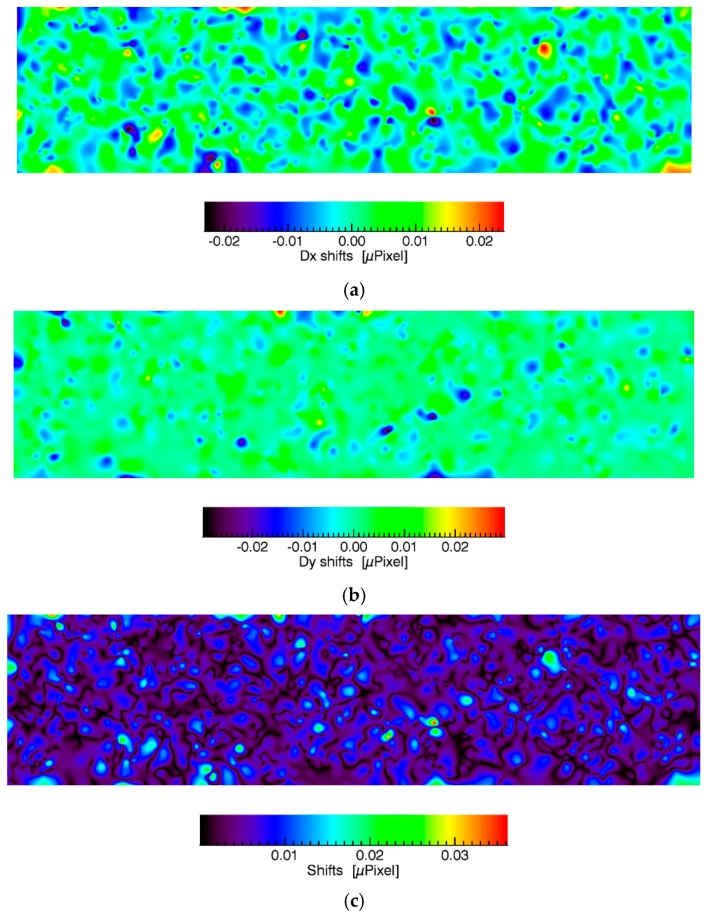

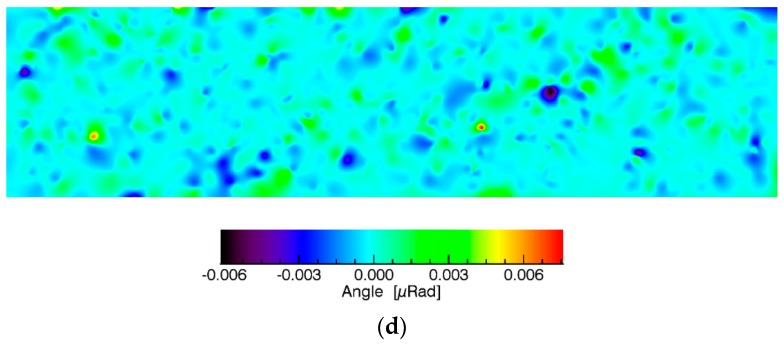
Interpolated local distortions that represent the accuracy of the geometry module for visual assessment. (**a**) Dx shifts, (**b**) Dy shifts (**c**) shifts in μpixel and angle distortions (**d**) in μRad. Please note that there are no significant gradients at the displayed micro-unit scale.

Table 3 indicates that the geometric distortion of the local SIFT windows between VNIR and SWIR is micro-pixel and micro-radiant precise. Additionally, the MSSIM between VNIR and SWIR was improved from 78% to 97%. A comparison of locally shifted windows for the whole image content is not possible, because only high contrast regions are usable. The matching around SIFT points was proven as stable in numerous applications. However, any of the criteria used here for evaluation can be subsequently utilized after the measurement as a quality control step. Therefore, it is also necessary to provide an image content covering an overview of potential distortions. For this, all computed distortions were interpolated using thin plate splines [32] and then visually and subjectively evaluated with regard to potential, dominating gradients. The results for the interpolation are shown in the following Figure 9.

**Figure 10:**
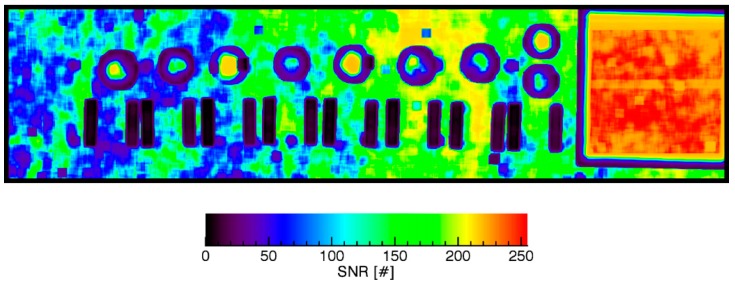
Image SNR variations due to scattering, albedo and illumination gradients.

The subjective evaluation of all Figure 9a–d indicates that there aren’t any extensive, dominating gradients in the micro-unit scale. It is concluded that the SIFT algorithm robustly selects high contrast regions that supports the assessments of local distortions. The number of outliers appears to be low. It also appears that the impact of the albedo on the fitting is low as long as the local contrast is high enough. As given by Figure 6c potential SIFT points were found everywhere in the images if the image SNR is high enough. The image SNR is impacted by a variety of factors. The most important factors are the temporal stabilities of the light source and of the sensor sensitivity. As those factors may non-linearly vary the estimation of the image SNR is not trivial and can be considered as an own subject of research. However, we applied a straightforward, simplified approach to retrieve the image SNR similar to the approach proposed by Gao et al. [33]. First, a copy of the image is smoothed using a 9 × 9 boxcar filter (moving average). Then, the local image content of a 16 × 16 pixel moving window is extracted from the smoothed and original image content. The averages of each window of the smoothed image and the standard deviations of each window of the difference between the smoothed and original image are computed. Then, the ratios between the averages and standard deviations are computed. The result is given in Figure 10.

This was performed for band 152 of the VNIR. The results for the SWIR are equivalent and have a MSSIM similarity of about 99% to those of the VNIR. Comparing the SIFT point distribution of the Figure 6c) with the SNR map of Figure 10 shows that SIFT point location is not primarily bound to the image SNR if the image SNR and the contrast are high enough. It was not possible in this work to estimate the minimum required SNR for SIFT based matching. However, the SNR map of Figure 10 reveals some other interesting details. The SNR of bright to dark powder samples is on average higher than the background. The highest SNR is retrieved on the reference plate. There is a slight SNR increase from the left to the right that might be caused by volume scattering from the reference plate as low variant albedo superimposition. The SNR on the reference plate is about twice the SNR of the low reflecting background. In summary the geometric module provides high precision for this exemplary setup and enables a variety of applications.

### 3.2. Spectrometry

The analysis quality highly depends on the quality of the at-surface reflectance retrieval, because often a statistical comparison between unknown image spectra and a known library is used as the basis for analyses, which requires a foregoing, efficient suppression of illumination and sensing geometry related signal variation. Two types of reflectance can be selected as the basis for comparison —absolute and relative reflectance. Relative reflectance can be retrieved if the reflectance retrieval utilizes a wavelength independent, averaged reflection coefficient of the reference plates for the irradiance estimation. Absolute reflectance can be retrieved if the full absolute reflectance spectrum of the reference plate is used for the irradiance estimation. It depends on the application, which mode should be selected. In this work, we use absolute reflectance, because its logarithmic inverse—absorbance—can be related to geochemically assessed concentrations with regard to the Beer-Lambert law. Therefore, the absolute reflectances of the spectral library of the reference plates were not averaged to relative reflectance. However, the following Table 4 clearly shows that the reflectance spectra of the ROI was highly precise derived.

The region growing improves the numerical modelling of the spatial illumination in general, because a spatially broader image extent is used for polynomial modelling. At the same time the spectral deviation of the reference plate increases that may have a negative impact on the spectral normalisation. This could be caused by several factors and may vary per scan. Potential factors are spectral superimposition of the plate border spectra by the spatially adjacent neighbourhood, increased contribution of noise, micro shadowing effects of the plate surface, dust, particles etc. However, the error is still low and around 1% which is highly acceptable for most applications. The third objective measure is the spectral variation which increases if any illumination trends remain, plates were not perfectly ‘spectrally’ clean and adjacency effects have occurred. The achieved variation of 2% clearly shows that there are still signal impacts, but this might be also insignificant for the most applications. In sum, all three measures show a high quality.

The subjective measures relate the spectra of different mineral standards measured with 3 different spectrometers—point spectrometer ASD, the imaging spectrometer HySPEX and the laboratory spectrometer Perkin Elmer. The results are given in the following Table 5.

With regard to the results of Table 6 the overall accuracy between the spectrometers significantly varies and depends on the individual physical reflection properties of the sample. Additionally, all spectrometers utilize completely different spectroscopic techniques that must provide different results (ASD bare fiber optics, HySPEX push broom scanning and Perkin Elmer integrating sphere), although this is an ongoing discussion in remote sensing. However, the overall accuracy for the full spectrum is relatively high except for calcite and pyrophyllite. Therefore, both minerals have special optical properties, which leads to different spectra. The reason is unknown and shall be investigated in further research. If the similarity is ranked as performed in the last row of Table 6, then the ASD and the HySPEX provide more equivalent spectra then the ASD and the Perkin Elmer or the HySPEX and the Perkin Elmer. This shows that point or single spot spectrometers like the ASD and the Perkin Elmer can provide significantly differing spectra and that spectra should be only compared if they originate from the same device. Advanced spatially and spectrally adaptation or cross-calibration can lead to comparable sensor responses [34]. However, different spectroscopic measurements provide different results for the same sample. Most analysis approaches only utilize spectral absorption features, which require a foregoing continuum removal. This was also performed and the evaluation was repeated using continuum removed absorption features. The results are given in the following Table 6.

**Figure 11:**
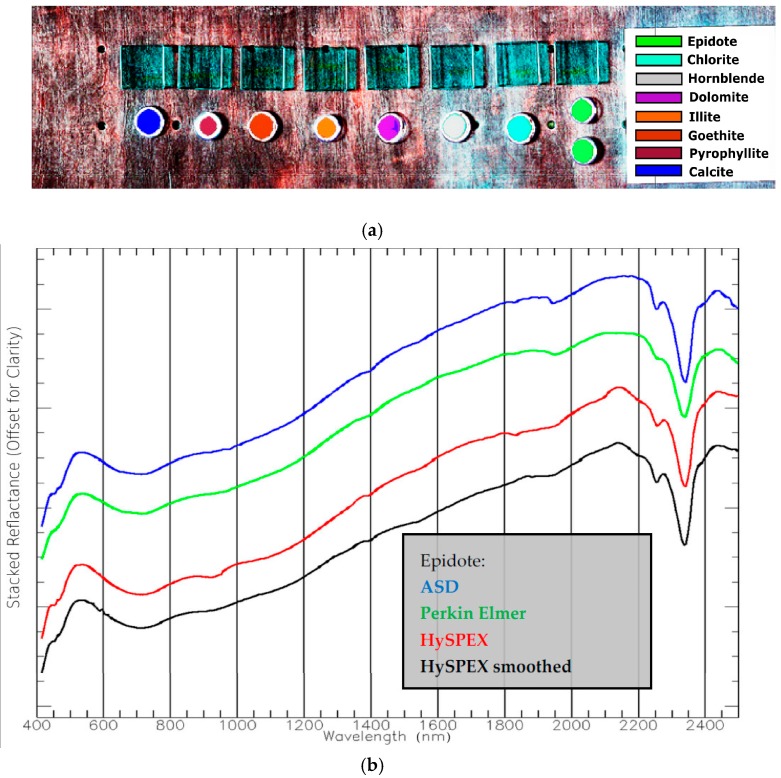
(**a**) False colour HySPEX image (Red 2210.02 nm, Green 800.295 nm, Blue 651.19 nm) overlain with classification of EnGeoMAP [1], (**b**) Epidote spectra from different spectrometers—‘HySPEX’ spectrum as spatial average of 10 × 10 pixel spectra, ‘HySPEX smoothed’ Gaussian smoothed spectrum (black).

Although the similarity rank for the ASD and the Perkin Elmer changed the ASD and the HySPEX achieve the highest rank again. The overall accuracy significantly decreased and even more underlines that different spectrometers provide different results. This is also valid for the reference plate. Calcite shows the highest deviation which supports the foregoing optical property assumption. It follows from this that if any spectral analysis is performed the incorporation of spectra from the same sensor may provide better results than from a different sensor. A realization of this is not state-of-the-art in remote sensing and, hence, may lead to varying results. Nevertheless, the HySPEX scan was also analysed with the EnGeoMAP approach of Mielke et al. [1] using a spectrally resampled ASD mineral standard library. The classification and sample spectra results are given in the Figure 11.

The results of Figure 11 clearly indicate for the selected example that the whole processing chain gives high quality results. The classification result for the dolomite sample contains false-positive identifications of calcite which is spectrally similar. The spectra of Figure 11 show that the imaging spectra is also similar to those derived from field and lab spectrometers. However, there are remaining differences which primarily originate from the different measurement principles. The difference between the spatial HySPEX average (green line) and the spectrally smoothed HySPEX spectrum (band isotropic Gaussian filter, black) is significant.

This difference in the spectrum may originate from the micro-shadows of the homogenized, not compacted powder sample, the sample holder material, variation of the grain illumination angle. However, the Gaussian smoothing preserves the absorption features better than the spatial binning. The preservation is a function of the filter type, filter width and filter strength. We selected in our tests an isotropic Gaussian filter with a FWHM of two bands for which we experienced overall good results in all laboratory applications if smoothing is necessary. This can be necessary if the selected integration time is relatively low compared to the optimal integration time for dark materials and the sample consists of both very dark and very bright materials such as Banded Iron Formations (BIF).

## 4. Concluding Remarks

The proposed approach can be utilized for a broad set of applications and is not limited to the HySPEX sensor. The overall geometric and spectral performance is constantly high and might be good enough for geoscientific applications. The achieved results clearly show that different sensors provide different spectra of the same material due to different sensing principles and related signal impacts. Therefore, a direct comparison is limited. Contrary, the low deviation between the ASD and the HySPEX measurements of the reference plate as a Lambertian diffuser indicate that the proposed chain is highly precise and that imaging spectrometers can provide highly qualitative spectra compared to established points spectrometers as those from ASD or Perkin Elmer. The capacity of imaging spectrometers to measure a high quantity of samples in a short time compared to field or laboratory spectrometers offers more applications. Additionally, spectra from imaging spectrometers are ‘more similar’ to those derived from airborne campaigns using the same system than those derived from field spectrometers. This may lead to better results for successive airborne image spectra adjustments like the broadly applied Empirical Line approach. Therefore, cross-calibration campaigns of the same sensor improve the overall accuracy and lead to better results, but not in the case of different sensors and/or sensing principles.

## Figures and Tables

**Table 1:** Specification of the imaging spectrometer system HySPEX VNIR/SWIR (from manufacturer online resource http://www.hyspex.no/products/disc.php accessed on 14 June 2017).

Type	VNIR 1600	SWIR 320 m-e
Spectral Range	0.4–1.0 μm	1.0–2.5 μm
Spatial Pixels	1600	320
F-number	F2.5	F2.0
FOV across track	17°	13.5°
Pixel FOV across/along track	0.18 mrad/0.36 mrad	0.75 mrad/0.75 mrad
Spectral sampling interval	3.7 nm	6 nm
Number of bands	160	256
Binning modes	2, 4, 8	-
Noise floor	40e	-
Peak SNR	>200	-
Dynamic range	1000	-
Digitization	12 bit	14 bit
Max frame rate	135 fps	100 fps
Sensor head weight.	4.6 kg	7.5 kg
Sensor head dim. (lwh in cm)	31.5 × 8.4 × 13.8	36 × 14 × 15.2
Sensor head pwr. cons.	~6 W	~100 W
FPA cooling temperature	-	~195 K
Smile (SSI, from Lenhard et al. [4])	0.11–0.19	0.13
Average keystone (ASI, from Lenhard et al. [4])	0.22	0.09
Laboratory illumination	Mercury high-pressure lamp 2× 900 W	

**Table 2:** Specifications of ASD 4 FieldSpec HiRes (from manufacturer online resource https://www.asdi.com/products-and-services/fieldspec-spectroradiometers/fieldspec-4-hi-res accessed on 14 June 2017) and PerkinElmer Lambda 950 [5].

Type	ASD FieldSpec 4 HiRes	Perkin Elmer Lambda 950
Spectral Range	0.35–2.5 μm	0.175–3.3 μm
Spectral sampling	1.4 nm @ 350–1000 nm; 1.1 nm @ 1001–2500 nm	UV/VIS 0.05 nm; NIR 0.2 nm
Wavelength accuracy	0.5 nm	UV/VIS 0.08 nm, NIR 0.3 nm
Illumination	Halogen	Deuterium (UV),Tungsten-halogen

**Table 3:** Exemplary results for SIFT point residuals.

Parameter/Criterion	Angle (μrad)	Scale (#)	Dx (μPixel)	Dy (μPixel)	MSSIM a Priori	MSSIM a Posteri
Mean 3σ	0.0004	1	0.004	0.002	0.78	0.97
σ	0.0006	0	0.003	0.003	-	-

**Table 4:** Objective evaluation of the spectral performance.

Parameter/Criterion	Spectral Deviation Reference Plate ROI (%)	Spectral Deviation Reference Plate Region Growing (%)	Spectral Variation Reference Plate (%)
Mean 3σ	0.015	1.023	2.125
σ	0.019	1.446	1.191

**Table 5:** Subjective evaluation of the spectral performance for the full spectrum.

Sample/Criterion	Spectral Deviation between ASD and Perkin Elmer (%)	Spectral Deviation between ASD and HySPEX (%)	Spectral Deviation between Perkin Elmer and HySPEX (%)
Epidote	0.107	0.785	0.723
Hornblende	0.097	0.375	0.477
Chlorite	0.129	0.536	0.545
Dolomite	0.036	0.450	0.409
Illite	0.127	0.555	0.599
Goethite	0.567	2.648	5.350
Pyrophyllite	3.039	7.859	6.466
Calcite	25.01	12.24	31.07
Reference plate		0.024	
Mean	3.64	2.83	5.71
σ	8.13	4.06	9.86
Similarity rank	2	1	3

**Table 6:** Subjective evaluation of the spectral performance for the continuum removed spectrum.

Sample/Criterion	Spectral Deviation between ASD and Perkin Elmer (%)	Spectral Deviation between ASD and HySPEX (%)	Spectral Deviation between Perkin Elmer and HySPEX (%)
Epidote	7.018	5.679	1.903
Hornblende	32.90	4.027	45.46
Chlorite	6.406	9.923	2.255
Dolomite	4.601	1.312	6.604
Illite	19.85	4.084	34.28
Goethite	3.408	1.121	6.238
Pyrophyllite	4.588	11.24	11.11
Calcite	59.18	20.30	28.20
Reference plate		1.879	
Mean	17.24	6.62	17.01
σ	18.52	5.91	15.56
Similarity rank	3	1	2

## Supplementary Materials

The following are available online at http://www.mdpi.com/1424-8220/17/8/1857/s1. The geo-corrected at-surface reflectance is enclosed as compressed ENVI BSQ zip file.
